# Abnormal hypophyseal and suspensor divisions in Arabidopsis *dcl1* embryos are not attributable to a single miR156-targeted *SQUAMOSA PROMOTER BINDING PROTEIN-LIKE (SPL)* gene, but likely involve redundant genetic pathways and/or modulation by genetic background

**DOI:** 10.1007/s00497-025-00531-3

**Published:** 2025-10-08

**Authors:** Andrea Tovar-Aguilar, Jianfei Zhao, Scott Poethig, Stewart Gillmor

**Affiliations:** 1 Unidad de Genómica Avanzada, Cinvestav, Irapuato, Guanajuato México; 2 Department of Biology, University of Pennsylvania, Philadelphia, PA USA; 3 Department of Biological Sciences, DePaul University, Lincoln Park Campus, Chicago, IL USA

**Keywords:** Pattern formation, Zygote, Embryo, microRNA, miR156, *SPL*, Arabidopsis

## Abstract

***Key message*:**

**Loss of** ***SPL10*** **and** ***SPL11*** **increases penetrance of abnormal phenotypes in** ***dcl1*** **embryos.**

**Abstract:**

The first division of the Arabidopsis zygote is asymmetric, resulting in an apical cell lineage that generates most of the embryo proper, and a basal cell lineage that produces the root meristem and the extraembryonic suspensor. Loss of function mutations in the microRNA processing enzyme genes *DICER-LIKE 1 (DCL1)* and *SERRATE (SE)* show cell division defects in the embryo proper, hypophyseal cell, and suspensor. Previous transcriptome analyses showed that the microRNA156-targeted transcription factor genes *SQUAMOSA PROMOTER BINDING PROTEIN-LIKE2 (SPL2)*, *SPL3* and *SPL11* were upregulated in both globular stage *dcl1* and *se* embryos, while *SPL10* was upregulated in *dcl1*. It was previously proposed that upregulation of *SPL10* and *SPL11* could explain some abnormal phenotypes in *dcl1* embryos. In this work, we used T-DNA and CRISPR-Cas9-induced loss of function alleles to further explore the function of *SPL2*, *SPL3*, *SPL10* and *SPL11* in early embryogenesis and their contribution to the *dcl1* phenotype. On their own, *spl2*, *spl3*, *spl10*, and *spl11* single mutants and an *spl10 spl11* double mutant showed no abnormal cell divisions in early embryogenesis. In the *dcl1/+* background, loss of function of *SPL2* or *SPL3* did not change the proportion of cell division defects in hypophyseal cells or suspensors observed in *dcl1/+*. Loss of *spl10* or *spl11* in *dcl1/+* resulted in a slight decrease or increase (respectively) in the penetrance of abnormal suspensor divisions in heart stage embryos, while the *spl10 spl11* double mutant caused a small increase in the penetrance of abnormal hypophyseal divisions in *dcl1* embryos. The differences between our results and previous studies are likely due to genetic redundancy of miR156-targeted *SPL* genes, variable environmental conditions or the effect of genetic background on the penetrance of the *dcl1* phenotype. In the future, analysis of higher order mutations in *SPL* and *MIR156* genes will help to better understand the role of these important developmental regulators in early embryo development.

**Supplementary Information:**

The online version contains supplementary material available at 10.1007/s00497-025-00531-3.

## Introduction

MicroRNAs are 20–22 bp non-coding small RNAs that regulate gene expression by targeting complementary mRNAs for transcript degradation and/or repression of translation via an ARGONAUTE protein such as ARGONAUTE1 (AGO1) (Voinnet 2009). miRNAs play roles in development such as regulation of leaf polarity, the timing of onset of leaf traits, and flowering (Dong et al. 2022). miR156, one of the most ancient miRNAs in plants (Cho et al. 2012), plays a crucial role in the timing of onset of leaf traits during vegetative development through targeting of transcripts of *SQUAMOSA PROMOTER BINDING PROTEIN-LIKE (SPL)* transcription factors (Xu et al. 2016). Previous RNAseq analyses of mutants in genes encoding the miRNA processing enzymes *DICER-LIKE 1 (DCL1)* and *SERRATE (SE)* showed that the miR156-targeted transcription factor genes *SPL2*, *SPL3* and *SPL11* have increased transcript levels in both *dcl1-5* and *se-1* embryos, while *SPL10* increased in *dcl1-5* embryos (Nodine and Bartel 2010; Armenta-Medina et al. 2017; Lepe-Soltero et al. 2017; Plotnikova et al. 2019). It was previously shown that some abnormal phenotypes in *dcl1* embryos were due to upregulation of *SPL10* and *SPL11* (Nodine and Bartel 2010). Consistent with this, expression of miR156-resistant versions of *SPL10* and *SPL11* led to abnormal divisions in the hypophyseal cell and its derivatives (Nodine and Bartel 2010; Plotnikova et al. 2019). We set out to validate and extend these results using the *dcl1-5* allele in the Columbia (Col) ecotype, novel CRISPR alleles of *SPL10* and *SPL2* in Col, and previously reported T-DNA alleles of *SPL3* and *SPL11* that were induced in the Wassilewskija (Ws) ecotype but which we introgressed into an isogenic Col background before analysis.

## Materials and methods

We used the *dcl1-5* null allele in the Columbia (Col) ecotype (ABRC stock number CS16069) which has a T-DNA insertion in the first exon (Schauer et al. 2002) and has been studied by Nodine and Bartel (2010) and Armenta-Medina et al. (2017). All *SPL* alleles used were in the Col ecotype and have been reported by Xu et al. 2016; except for *spl2-2*, a novel CRISPR-Cas9 allele containing an A nucleotide insertion after codon 33 of the *SPL2* coding sequence, just upstream of its SBP domain. This insertion causes a frameshift resulting in premature stop codons located four and seven codons downstream of the insertion site. The sequences around this insertion are: wild type: 5’-aga aag cta aaa cca atg gag tgg gaa att gat gga-3’ and *spl2-2*: 5’-aga aag cta aaa cca atg *A*ga gtg gga aat TGA tgg att TGA-3’, where *A* denotes the insertion and TGA denotes the stop codons that result from the frameshift induced by the A insertion. *spl3-1* (Flag_173C12; ABRC stock number CS69782) and *spl11-1* (Flag_422H07; ABRC stock number CS69786) were generated in the Wassilewskija (Ws) ecotype by the Institute of Agronomic Research in Versailles, France (Samson et al. 2002), and introgressed 6 times into Col (Xu et al. 2016). Both T-DNA alleles have previously been shown to result in undetectable levels of mRNA for the corresponding genes (Xu et al. 2016). *spl10-2* was generated by CRISPR-Cas9 mutagenesis (ABRC stock number CS69785). *SPL10* (At1g27370) and *SPL11* (At1g27360) are located about 1 kb apart on Chromosome 1, making it nearly impossible to generate a double mutant by recombination. To produce an *spl10 spl11* double mutant, the *SPL10* gene in the *spl11-1* single mutant was targeted using CRISPR-Cas9 to produce the *spl10-3 spl11-1* double mutant (ABRC stock number CS69791). All genotyping primers used are listed in Table S1.

As shown in Table 1, *spl2-2* and the *spl10-3 spl11-1* double mutant did not show abnormal phenotypes in early embryogenesis. To determine if these alleles conditioned a discernable phenotype at other stages of the life cycle, we determined their effect on the onset of leaf abaxial trichome formation during vegetative development. Wt Col produced an average of 5.2 leaves without abaxial trichomes (*n* = 47), *spl10-3 spl11-1* produced an average of 5.3 leaves without abaxial trichomes (*n* = 24), *spl2-2* produced an average of 6.3 leaves without abaxial trichomes (*n* = 41), and *spl2-2 spl10-3 spl11-1* plants produced 7.3 leaves without abaxial trichomes (*n* = 24). Statistical analysis using a Bonferroni corrected t-test showed that *spl2-2* had significantly later trichome production than wt and (*p* = 1.1e^−7^) and *spl10-3 spl11-1* (*p* = 4.2e^−7^), while the effect of *spl2-2* on trichome production was significantly enhanced in the *spl2-2 spl10-3 spl11-1* triple mutant (*p* = 9.1e^−6^). The effect of *spl2-2* on vegetative development and the enhancement of *spl2-2* by *spl10-3 spl11-1* demonstrates that these allelic combinations produce a loss of function in the corresponding gene products.

**Table 1:** Quantification of hypophyseal, suspensor and seed defects in Wt Col, *dcl1* and *spl* genotypes

	Abnormal division of hypophysis	Abnormal division of suspensor cells	
	3 dap	5 dap	7 dap
Wild Type Col	0%	0%	0%
	*n* = 664	*n* = 508	*n* = 627
*dcl1-5/+*	24.1%***	27.6%***	25.3%***
*Fisher’s p*	3.71e^−51^, *n* = 568	7.05e^−52^, *n* = 691	1.38e^−60^, *n* = 615
*spl2-2*	0%	0%	0%
*Fisher’s p*	1, *n* = 604	1, *n* = 661	1, *n* = 693
*spl3-1*	0%	0%	0%
*Fisher’s p*	1, *n* = 502	1, *n* = 409	1, *n* = 556
*spl10-2*	0%	0%	0%
*Fisher’s p*	1, *n* = 511	1, *n* = 464	1, *n* = 492
*spl11-1*	0%	0%	0%
*Fisher’s p*	1, *n* = 616	1, *n* = 389	1, *n* = 534
*spl10-3; spl11-1*	0%	0%	0%
*Fisher’s p*	1, *n* = 500	1, *n* = 507	1, *n* = 407
*dcl1-5/+; spl2-2*	27.8%	30.6%	28.1%
*Fisher’s p*	0.1, *n* = 417	0.3, *n* = 418	0.35, *n* = 419
*dcl1-5/+; spl3-1*	27.1%	30.4%	24.48%
*Fisher’s p*	0.29, *n* = 409	0.08, *n* = 450	0.25, *n* = 486
*dcl1-5/+; spl10-2*	27.9%	25.6%**	27.3%
*Fisher’s p*	0.15, *n* = 541	2.14e^−3^, *n* = 515	0.9, *n* = 652
*dcl1-5/+; spl11-1*	28.5%	28.6%*	29.7%
*Fisher’s p*	0.1, *n* = 533	4.77e^−2^, *n* = 328	0.49, *n* = 313
*dcl1-5/+; spl10-3; spl11-1*	31.7%**	27.3%	27.6%
*Fisher’s p*	7.95e^−3^, *n* = 428	0.94, *n* = 461	0.41, *n* = 525

All data are from hand pollinations. Plants were emasculated and pollinated two days later. For Fisher’s two-tailed test for significance of differences in penetrance between genotypes: *dcl1-5/+*, *spl* single mutants and *spl10* *spl11* double mutants were compared with Wt Col; *dcl1-5/+* *spl* double and triple mutants were compared with *dcl1-5/+*. dap, days after pollination. ***, p < 0.001; **, p < 0.01; *, p < 0.05

Seeds were sown on soil and stratified at 4 °C for 3 days, then grown under greenhouse conditions. To generate multiple mutant combinations, *dcl1-5/+* plants were emasculated and pollinated by *spl2-2*,* spl3-1*,* spl10-2*,* spl11-1*, or *spl10-3 spl11-1*. F2 plants were genotyped by PCR for all *spl* alleles while the *dcl1-5* allele was genotyped based on its embryo phenotype and by PCR. For analysis of embryo phenotypes, greenhouse-grown plants were emasculated and then placed into a growth chamber at 22 °C under 16 h light/8hr dark conditions at a light intensity of 100 µmol m^−2^s^−1^PAR, pollinated 2 days later, and returned to the growth chamber. For DIC microscopy, fresh embryos were cleared in Hoyer’s solution (Anderson 1954) for 24 to 48 h at 4 °C, and samples were observed on a Leica DM6000B Nomarski microscope.

## Results and discussion

Both the *DCL1* and *SE* genes encode proteins that are essential for processing of pre-microRNA mRNA transcripts into functional miRNAs, and thus both are expected to result in an absence of miRNAs (Schauer et al. 2002; Yang et al. 2006). Loss of function alleles of *DCL1* and *SE* show similar phenotypes in early embryogenesis (Schwartz et al. 1994; Grigg et al. 2009; Nodine and Bartel 2010; Seefried et al. 2014; Armenta-Medina et al. 2017), presumably due to lack of function of the same miRNAs and consequent misregulation of most of the same miRNA-targeted genes. Transcriptome analyses of globular stage *dcl1-5* embryos (Nodine and Bartel 2010) and globular stage *se-1* embryos (Armenda-Medina et al., 2017; Lepe-Soltero et al. 2017) found the miR156-targeted transcription factor genes *SPL2*, *SPL3* and *SPL11* to be upregulated in both genotypes, and *SPL10* to be upregulated in *dcl1-5*. While *SPL2* and *SPL3* showed modest increases (less than two-fold in *se-1* and *dcl1-5*), *SPL10* increased almost 30-fold in *dcl1-5* and *SPL11* increased more than 4-fold in both *se-1* and *dcl1-5* (Nodine and Bartel 2010; Armenta-Medina et al. 2017; Lepe-Soltero et al. 2017). A subsequent analysis of miRNA targeted mRNAs found *SPL2* cleavage products to be enriched in globular stage embryos (Plotnikova et al. 2019). These results led us to test the hypothesis that the division defects in *dcl1* hypophyseal and suspensor cells might be due to upregulation of these four individual *SPL* genes and to revisit the hypothesis that combined upregulation of *SPL10* and *SPL11* contributed to the *dcl1* phenotype (Nodine and Bartel 2010).

**Fig. 1:**
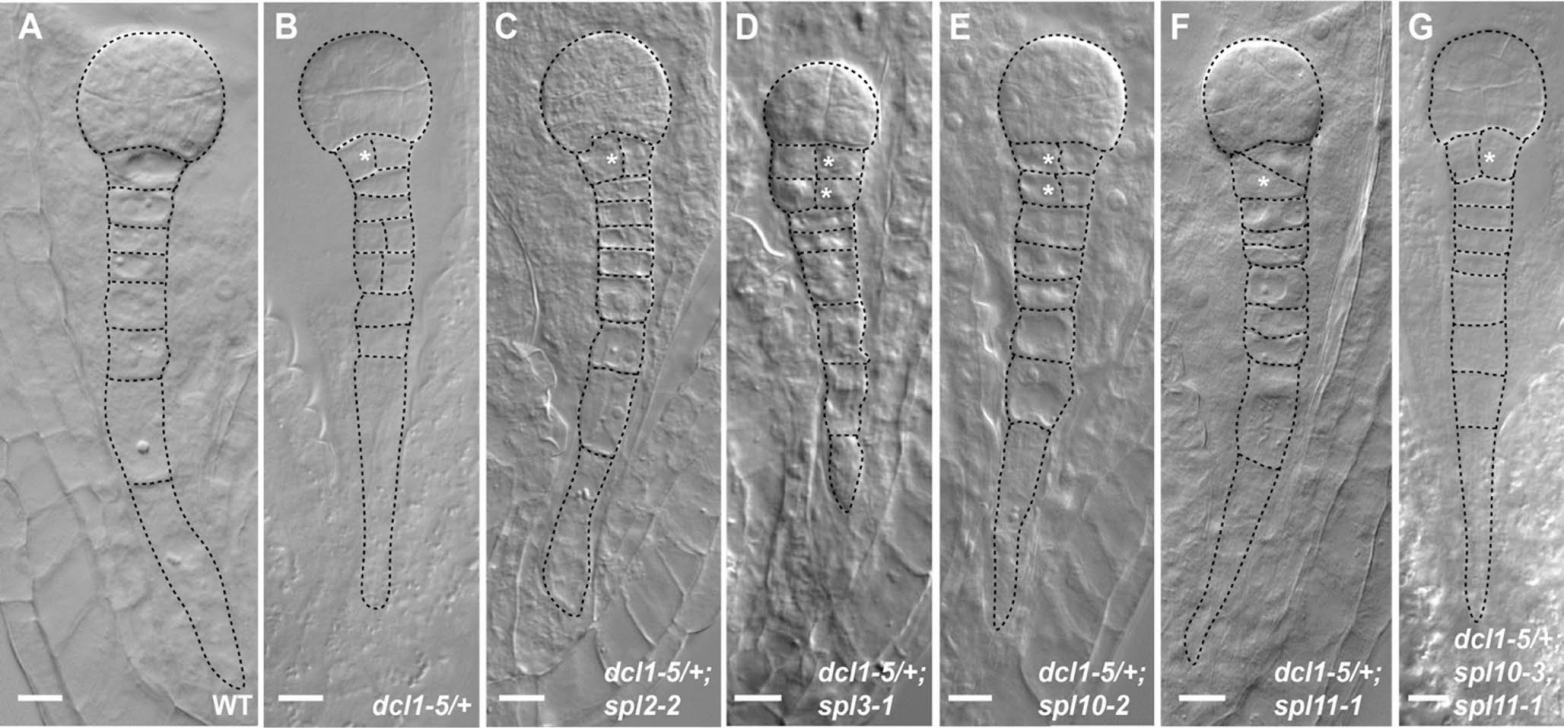
Abnormal divisions of the hypophyseal cell and suspensor in embryos of *dcl1/+* plants and in embryos of *dcl1/+* in the *spl2*, *spl3*, *spl10*, and *spl11* single mutant backgrounds and in the *spl10 spl11* double mutant Embryos obtained from the indicated genotypes are shown at 3 days after pollination. Outlines of cells have been traced with a dotted line to make cell division patterns clearer. Abnormal (oblique or vertical) divisions of the hypophyseal cell are marked with an asterisk. See Table 1 for quantification of penetrance of phenotypes. Scale bar, 10 μm

The *dcl1-5* null allele is embryo lethal, so all analyses of *dcl1* phenotypes were carried out using seed from plants heterozygous for *dcl1-5* (*dcl1-5/+*), which segregate ~ 25% abnormal phenotypes in globular stage and later embryos (Nodine and Bartel 2010; Armenta-Medina et al. 2017). The most penetrant and easily distinguishable phenotypes of *dcl1* mutants are abnormal divisions in the hypophyseal cell at the globular stage and in the suspensor cells at the heart stage (Nodine and Bartel 2010). We scored the penetrance of these phenotypes in embryos of hand self-pollinated plants of wild type (wt) Col and *dcl1-5/+* plants at 3, 5 and 7 days after pollination (dap) (Fig. 1 and Figure S1; Table 1). In siliques of *dcl1-5/+* plants at 3 dap (wt globular stage), 24.1% (*n* = 568) of embryos showed abnormal divisions of the hypophyseal cell, while no abnormal divisions were observed in embryos of wt Col plants (Fig. 1A and B; Table 1). At 5 dap (wt heart stage), 27.6% (*n* = 691) of embryos produced by *dcl1-5/+* plants showed abnormal divisions of the suspensor while no abnormal divisions were observed in embryos from wt plants (Figure S1A and B; Table 1). At 7 dap (wt early bent cotyledon stage), 25.3% (*n* = 627) of embryos in *dcl1/+* plants were arrested at the globular stage with overproliferating suspensors; embryos of wt plants no longer have suspensors at this stage (Figure S1H and I; Table 1).

To determine if loss of *SPL2*, *SPL3*, *SPL10* and/or *SPL11* could ameliorate abnormal hypophyseal or suspensor cell divisions in embryos of *dcl1-5/+* plants, we examined the phenotype of embryos at 3, 5 and 7 dap segregating in siliques of plants that were heterozygous for *dcl1-5* and homozygous for single *spl* mutants or the *spl10 spl11* double mutant, as well as plants that were homozygous only for the single *spl* mutants and the *spl10 spl11* double mutant. For the hypophyseal cell, an abnormal division was scored as an oblique or vertical division which did not produce the characteristic lens-shaped cell. In the suspensor, divisions that were not perpendicular to the long axis of the suspensor were scored as abnormal. *spl2*, *spl3*, *spl10*, *spl11* and *spl10 spl11* plants did not show any cell division defects in the hypophyseal or suspensor cells (Table 1), as previously observed for *spl11*, *SPL10* RNAi lines, and the combination of *spl11* and the *SPL10* RNAi line (Nodine and Bartel 2010). The combination of either *spl2* or *spl3* with *dcl1-5/+* had no statistically significant effect on abnormal divisions in hypophysis or suspensor cells at 3, 5 or 7 dap (Fig. 1, Figure S1, Table 1). Combining *spl10* with *dcl1-5/+* caused a 2% decrease in abnormal divisions of the suspensor at 5 dap (27.6% in *dcl1-5/+* vs. 25.6% in *dcl1-5/+ spl10*; *p* < 0.01), while combining *spl11* with *dcl1-5/+* caused a 1% increase in abnormal suspensor divisions at 5 dap (27.6% in *dcl1-5/+* vs. 28.6% in *dcl1-5/+ spl11*; *p* < 0.05). Placing *dcl1-5/+* in the *spl10 spl11* double mutant background caused a 7% increase in the penetrance of abnormal divisions of the hypophysis at 3 dap (24.1% in *dcl1/+* vs. 31.7% in *dcl1/+ spl10 spl11*; *p* < 0.01) (Fig. 1, Figure S1, Table 1).

In contrast to our results, Nodine and Bartel (2010) found that *spl11-1* decreased the penetrance of hypophyseal phenotypes in embryos of *dcl1/+* plants from the expected 25% to 16.8%, and RNAi silencing of *SPL10* in the *dcl1−5/+ spl11-1* background further decreased the penetrance of hypophyseal defects to 8%. Phenotypic rescue of *dcl1-5* phenotypes by *spl11-1* combined with *SPL10-RNAi* was only observed at the globular stage; heart stage embryos showed the *dcl1* phenotype at full penetrance (Nodine and Bartel 2010). One explanation for the difference in penetrance of globular stage *dcl1* phenotypes in *SPL10 SPL11* loss of function backgrounds in our study compared with that of Nodine and Bartel (2010) is the genetic background of the *spl11-1* allele, which was induced in the Wassilewskija (Ws) background (Samson et al. 2002). While both our study and that of Nodine and Bartel used the *spl11-1* allele, we introgressed the *spl11-1* allele into Col by six outcrosses before undertaking our analyses. Nodine and Bartel (2010) did not specify whether *spl11-1* was introgressed into Col before crossing with *dcl1-5*. Given this, it is likely that their analyses of interactions between *dcl1-5*, *spl11-1* and *SPL10-RNAi* were carried out in a mixed Col-Ws ecotype background. Mixed ecotype backgrounds often decrease the penetrance of mutant phenotypes, and are known to alter gene expression in the embryo (Del Toro-De León et al. 2014; Alaniz-Fabián et al. 2022, 2025). Differing growth conditions may also explain the increased penetrance of *dcl1* phenotypes in our laboratory: Armenta-Medina et al. (2017) observed *dcl1* phenotypes beginning in the zygote, while Nodine and Bartel (2010) observed *dcl1* phenotypes only later, at the octant stage.

In summary, we found that, in an isogenic Col background, introducing an *spl10* null mutant into *dcl1-5/+* caused a small decrease in abnormal divisions in *dcl1* embryos, introducing an *spl11* loss of function into *dcl1/+* caused a small increase in abnormal divisions, and introducing both *spl10* and *spl11* mutants into *dcl1/+* caused a 7% increase in penetrance of abnormal hypophyseal divisions. Meanwhile, introduction of *spl2* and *spl3* mutants into *dcl1/+* had no effect on the *dcl1* phenotype. Thus, of these four *SPL* genes, our results show a small role for only *SPL10* in abnormal hypophyseal divisions in *dcl1* mutant embryos. As mentioned above, a previous analysis found that loss of *SPL10*, *SPL11* and *SPL10* and *SPL11* together led to strong, but transient, suppression of the *dcl1* phenotype at the globular stage (Nodine and Bartel 2010). Expression of miR156-resistant versions of *SPL10* and *SPL11* was also shown to result in abnormal divisions of the hypophyseal cell (Nodine and Bartel 2010; Plotnikova et al. 2019). Although miR156-resistant versions (i.e. dominant alleles) of these two *SPL* genes cause abnormal divisions in the hypophyseal cell and its derivatives, this does not conclusively demonstrate that abnormal hypophyseal divisions in *dcl1* embryos are due to altered regulation of *SPL10* and *SPL11* in *dcl1/+* plants, because dominant alleles demonstrate what a gene can do, not necessarily what it does in its normal biological context. In the future, it will be informative to compare the effect on the *dcl1* phenotype of the *spl10* and *spl11* alleles used in our study with the *spl11* and *SPL10* RNAi lines used by Nodine and Bartel (2010) in the exact same experimental conditions, as the contrasting results might be due to different growth conditions.

## Conclusion

Preglobular and globular embryo transcriptome datasets of Arabidopsis suggest that *MIR156A*, *MIR156B* and *MIR156C* genes are the primary source of miR156 in early embryos (Armenta-Medina et al. 2017; Lepe-Soltero et al. 2017; Plotnikova et al. 2019; Gao et al. 2022). As for miR156-targeted *SPL* genes, in addition to *SPL2*, *SPL3*, *SPL10* and *SPL11*, early embryo transcriptomes also show expression of *SPL6*, *SPL9*, *SPL13A*, *SPL13B* and *SPL15* (Plotnikova et al. 2019; Zhao et al. 2019). In the future, the hypothesis that loss of miR156 causes abnormal phenotypes in early embryos could be more precisely tested by examining mutants in *MIR156A MIR156B* and *MIR156C*, thus avoiding the pleiotropic effects of other miRNAs lost in early *dcl1* embryos such as miR160, miR165/6, miR167 and miR319 and the consequent upregulation of their transcription factor target genes *AUXIN RESPONSE FACTOR (ARF) ARF17* and *ARF8*; *PHABULOSA (PHB)*; and *TEOSINTE BRANCHED1-CYCLOIDEA-PROLIFERATING CELL NUCLEAR ANTIGEN FACTOR4 (TCP4)* (Nodine and Bartel 2010; Plotnikova et al. 2019). Conversely, higher order combinations of mutants in the many *SPL* genes expressed in embryos would provide insight into the consequences of loss of *SPL* function in early embryos. Given the importance of miR156 and *SPL* transcription factors in post-embryonic stages of the life cycle (Poethig and Fouracre 2024), it is likely that miR156 and some of its targets play important roles in early embryogenesis.

## Supplementary Information

Below is the link to the electronic supplementary material.

Supplementary Material 1
