# Supplementary material for: Abnormal hypophyseal and suspensor divisions in Arabidopsis dcl1 embryos are not attributable to a single miR156-targeted SQUAMOSA PROMOTER BINDING PROTEIN-LIKE (SPL) gene, but likely involve redundant genetic pathways and/or modulation by genetic background
Source: Plant Reprod. 2025 Oct 8;38(4):20. doi: 10.1007/s00497-025-00531-3 (PMC12507998; doi:10.1007/s00497-025-00531-3)
Supplement: Supplementary file 1 — Supplementary Material 1 [file 497_2025_531_MOESM1_ESM.docx]

**Supplementary Material**


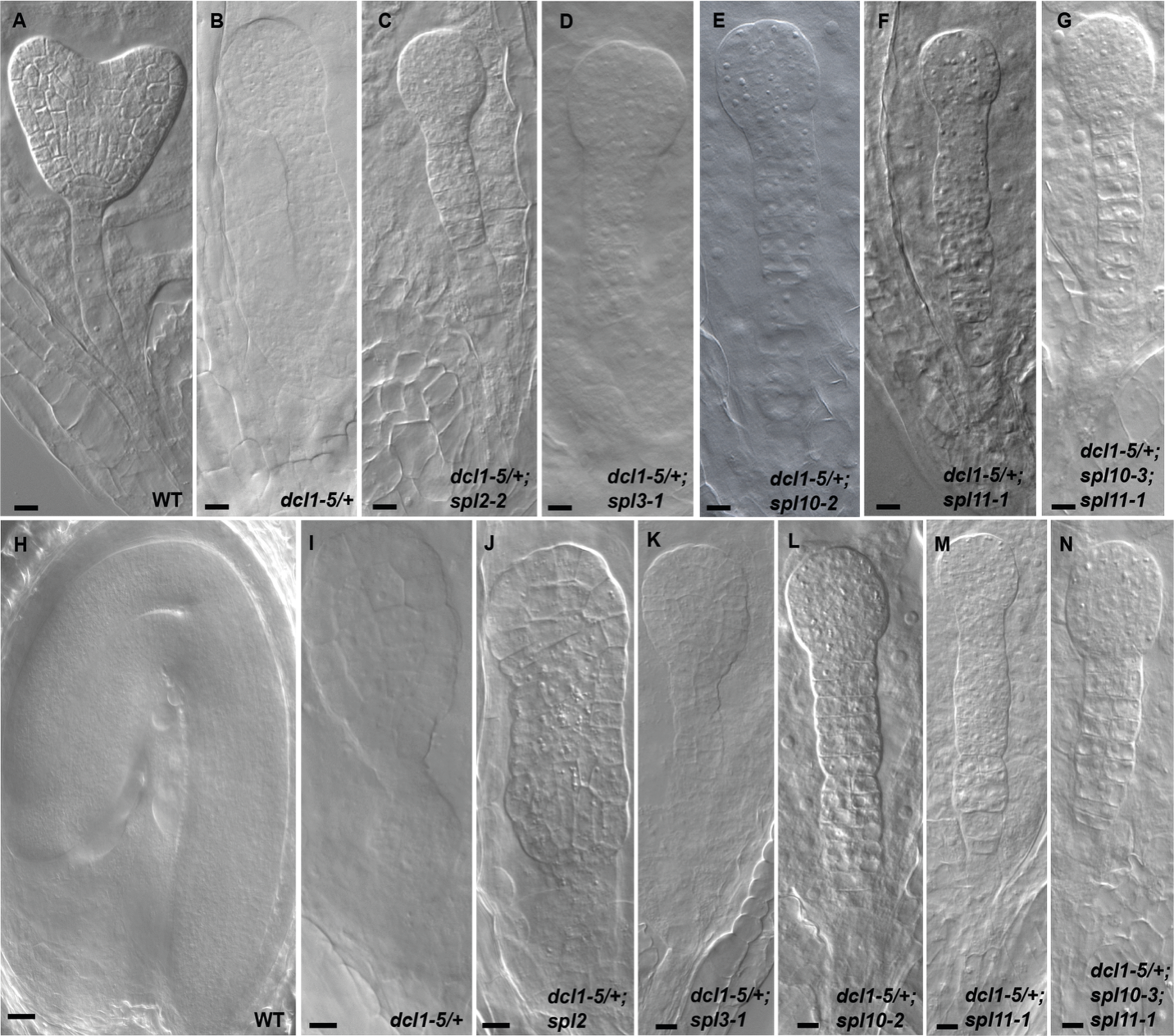


**Figure S1. Embryos from plants of Col wt, *dcl1-5/+*, *dcl1-5/+ spl2-2*, *dcl1-5/+ spl3-1*, *dcl1-5/+ spl10-2*, *dcl1-5/+ spl11-1* and *dcl1-5/+ spl10 spl11* at 5 and 7 dap.** Scale bar, 10µm.

| **Table S1. Oligonucleotide primers used in this study** | | |
| --- | --- | --- |
| Sequence | Primers 5’ to 3’ | PCR products in plant genotyping |
| Fw- *spl2-2* | CTAGAAAGCTAAAACCCATG | PCR product is 192 pb, digestion with NcoI gives a wt allele 171bp + 20bp and *spl2-2* can not be digested. |
| Rv- *spl2-2* | CCAGTTTCGGATGTAAAATTGTG |  |
| LP- *spl3-1* | TGGTCCAACAACTTAAGCACC | PCR WT product is 1112 bp (LP + RP), and T-DNA is 800 bp (LB4 + RP). |
| RP- *spl3-1* | TGGGAATCAAACGTGACTAGG |  |
| Fwd- *spl10-2* | AGGACAAACGATGCAATCTTG | PCR WT band is 300 bp and *spl10-2* 250 bp. |
| Rev- *spl10-2* | TTTTCTTCCGAGCAACAACAG |  |
| RP- *spl11-1* | AAATTGATGGCTGTGAACTGG | PCR WT product is 900 bp (LP + RP), and T-DNA is 500 bp (LB4 + RP). |
| LP- *spl11-1* | GAGCACGGTGGATATCTTGAG |  |
| Fw *dcl1-5* | CTGCAAACAACAACTTCAAAATTGGCG | PCR WT product is 1651 bp (Fw *dcl1 +* Rv *dc1l-5*) and T-DNA allele is 1200 bp (Fw *dcl1-5* + LB1). |
| Rv *dcl1-5* | CCTTTAAATTAACAGGCGAAGCAGTC |  |
| LB1 | GCGTGGACCGCTTGCTGCAACT |  |
| LB4 | CGTGTGCCAGGTGCCCACGGAATAGT |  |
